# Energy management system in smart buildings based coalition game theory with fog platform and smart meter infrastructure

**DOI:** 10.1038/s41598-023-29209-4

**Published:** 2023-02-04

**Authors:** Mohammed A. Saeed, Abdelfattah A. Eladl, Bilal Naji Alhasnawi, Saad Motahhir, Anand Nayyar, Mohd Asif Shah, Bishoy E. Sedhom

**Affiliations:** 1 Electrical Engineering Department, Faculty of Engineering, Mansoura University, Mansoura, 35516 Egypt; 2 Communications Engineering Department, Mansoura Higher Institute of Engineering and Technology, El-Mansoura, Egypt; 3 Department of Computer Technical Engineering, College of Information Technology, Imam Ja’afar Al-Sadiq University, Al-Muthanna, 66002 Iraq; 4 ENSA, SMBA University, Fez, Morocco; 5 Graduate School, Faculty of Information Technology, Duy Tan University, Da Nang, Viet Nam; 6 Department of Economics, Kebri Dehar University, 250 Somali, Ethiopia; 7 School of Business, Woxsen University, Kamkole, Sadasivpet, Hyderabad, 502345 Telangana India; 8 Division of Research and Development, Lovely Professional University, Phagwara, 144001 Punjab India

**Keywords:** Electrical and electronic engineering, Power distribution

## Abstract

This paper proposes a central energy management system (EMS) in smart buildings. It is based on the coalition method for optimal energy sharing between smart buildings. Game theory is applied to obtain an optimal allocation of the building's surplus energy on the deficient energy buildings using the Shapley value, which enables the unequal energy distribution based on the energy demand. The main objective is reducing energy waste while preserving the generation/demand balance. The fog platform with memory storage is applied, which handles all the measured data from the smart buildings through Wi-Fi-based communication protocol and performs the EMS program. The smart meter links the smart buildings with the fog-based EMS central unit. Two scenarios are implemented based on the difference between total deficient and surplus energy. Coalition game theory is applied for optimal surplus energy allocation on deficient buildings when the total energy surplus is lower than the total energy deficient. Also, there is a one-to-one relationship between the surplus and deficient building; if the surplus energy is larger than the deficit, the extra surplus energy is stored for further usage. The proposed EMS is applied and tested using a smart city with 10 buildings in the MATLAB program. A comparison between the result obtained with and without applying the proposed method is performed. The performance of the fog platform is introduced based on the run and delay time and the memory size usage. The results show the effectiveness of the proposed EMS in a smart building.

## Introduction

Many scientists agree that global warming poses the greatest threat to humanity. The United Nations Intergovernmental Panel on Climate Change has issued a warning despite the Paris Agreement. The world is not doing nearly enough to meet the goal of keeping the temperature increase below 1.5 °C^1^. There needs to be significant involvement from the building industry. On a global scale, it uses up more than a third of all energy produced, thirty percent of all greenhouse gases released, and forty percent of all natural materials^2^. The United States Energy Information Administration reported that commercial and residential buildings rely heavily on electrical appliances, lighting, heating, ventilation, and air conditioning (HVAC), making electricity the largest energy consumer in the building environment^3^. Reducing energy usage and the accompanying problems requires an in-depth comprehension of the building sector's energy consumption and environmental impact.

From this point, smart grids can manage energy generation, transmission, and distribution in real-time. It provides two-way communication between stakeholders, improving energy production, distribution, and consumption^4^. Information regarding energy usage in real-time is of the utmost importance to attain the energy conservation objective. This information allows for more accurate planning of how much energy will be used at various times throughout the day. It is also incredibly helpful for energy conservation and cost reduction to use alternative energy sources, such as renewable sources within buildings. Internet of things (IoT) is the modern term that emerged due to the tremendous development in smart grid technologies. When smart grids are combined with IoT, the result is a system that is extremely dynamic and highly efficient in energy distribution and consumption. This system exchanges information by using sensors and other functions that are functionally analogous to improve efficiency and functionality while consuming less energy^5^. The concept of smart grids began to be applied to new horizons, especially for electrical energy in buildings, to show the term smart buildings. Different smart energy devices and data analysis software are used to keep track of the environment and the energy consumption habits of building occupants to optimize building energy performance through automatic control that is central to the smart building technology paradigm^6^.

Multiple strategies have been developed to reduce energy usage in intelligent environments. Frequently, these challenges are phrased as optimization problems with objectives such as lowering the system's cost while ensuring the value of power flow constraints. Four main building energy management architectures are already in use. (A) Statistical models, (B) Architectures based on cloud computing, (C) Architectures based on smart meters, and (D) Architecture based on fog computing.

Training statistical models can forecast the energy consumption of different buildings. These methods are based on the predictions made by the trained models, which can be used to evaluate the actual effect of energy conservation measures, locate and solve problems, and incentivize people to reduce their energy use. Symbolic aggregate approximation (SAX) classifies the data of a building in several time windows, each of which displays a straightforward representation of a complicated, large dataset. The authors in^7^ offer a modification to the standard SAX method in which they use regression models to construct a time matrix for different energy values. Then, separate the aberrant changes, which can aid in detecting energy-related irregularities, allowing for early intervention to prevent building accidents. The authors in^8^ used generalized additive statistical modeling to predict gas consumption for two building datasets from two continents. Experiments were carried out on two distinct buildings; however, the quantity of findings from building one is restricted. In^9^, case-based reasoning and SCADA are applied to a society based on multi-agent systems (MAS). All of a building's energy use can be modeled by the MAS, thanks to the information it collects from various agents.

Fortunately, cloud computing, which is an on-demand, intermittent computing architecture, is emerging as a viable means of overcoming the aforementioned problems. For energy management in smart buildings, it is an essential resource for the full management of building-related information. It is a natural fit for building energy management in cases where most parameters are in real-time. Most cloud-based solutions aim to reduce energy use on the consumer end, but producers can reap savings by monitoring consumer habits and adjusting the output accordingly.

Swarm intelligence is integrated into fog nodes to evaluate the energy consumption of a smart house with sensors^10^. This device keeps constant tabs on how much power individual home appliances use, alerting the owner whenever something unexpected occurs. The authors provide comprehensive simulation results to prove that their method is effective and timely. Authors in^11^ use graph theory and clustering to characterize the temperature ranges within a building. To manage these extreme temperatures, an IoT slicing technique has been developed. With inputs from the building's control room temperatures, the application applies game theory to ascertain the status of each IoT node and make necessary adjustments. While the authors make several intriguing suggestions for regulating temperatures, they fail to provide the extensive experimental findings necessary to verify the algorithm's effectiveness. The authors in^12^ employ smart meters to keep tabs on several different Tunghai University buildings and then run all of that data via the renowned Hadoop system on a cluster of computers in real-time. Hadoop also uses distributed storage, which increases data capacity, to store sensory data. Authors in^13^ provided an automated cloud-based method that generates an intelligent structure pattern that can be utilized on several buildings. Still, no actual data indicates that the proposed strategy can also optimize energy on multiple buildings. To make a building "smart," the authors in^14^ choose a modest approach and automate just one wall plug. Over time, various outlet-related statistics are collected and made available to users via an Android app that queries a cloud database.

Fog computing is an overlay on the cloud computing infrastructure that improves upon its core competencies by adding layers of protection, redundancy, and scalability for data transmission and storage^15^. The authors in^16^ also use a fuzzy-fog model, with the fog layer as a bridge between the cloud and the edge layers. Fuzzy logic is used at the fog layer to process the data acquired from the sensors via the edge layer. To further demonstrate the efficacy of their fuzzy-fog architecture concerning energy savings, the authors run comprehensive simulations using both a virtual and an actual smart house. They reported that the fog-based concept must be tested on multiple residences or an entire building, which could dramatically alter the energy efficiency values attained by the proposed method. Authors in^17^ design a fog-based game-theory strategy in which smart homes coordinate their energy consumption schedules to reduce everyone's energy bills. Introducing the fog layer, which is faster than cloud computing, allows for more efficient scheduling of energy resources. The authors in^18^ expanded the standard TCP/IP paradigm by adding two new layers: a fog-computing layer and a cloud-computing layer. Because of the reduced latency and real-time processing that these layers provide, a smart home can adapt to its residents' needs in various ways. Sensors are installed in the fog computing layer to centralize data collection at the periphery. Big data that is too large to process locally is sent to the cloud computing layer. Using temperature, power, and motion sensors data, the authors simulated the proposed algorithm on a smart meter at Alborg University.

Different meter designs are suggested for consumers and power distributors. In^19^, the authors design a modular smart meter framework for estimating the power needs of tiny buildings. Modifications were made to the open system interconnection layer model to illustrate the Zigbee connection between end users, service providers, and the cloud. The data collected by the meter is then used to illustrate how people typically manage their energy consumption. The authors in^20^ built a smart meter based on fuzzy logic to reduce the money customers spend on electricity while increasing their security level. LCD monitor, buttons, a power supply, and serial port modules are incorporated into the design, along with a Wi-Fi connection. To regulate the users' energy usage and production, the developed hardware incorporates fuzzy rules into its design. The results illustrate the difference in the average daily load with and without incorporating the suggested meter. The authors in^21^ used K-means clustering to arrange the buildings following their power usage; nevertheless, the smart meter is just used for its name, and no actual discussion or detail is offered. Table 1 summarizes all the above techniques, explaining the pros and cons of each one.

**Table 1:** A Summary of energy management techniques in smart buildings-based solution.

References	Year	EMS method	Advantages	Disadvantages
^7^	2018	Symbolic Aggregate Approximation (SAX)	The information is gathered from two distinct locations	Results only include electrical energy demand
^8^	2018	Generalized Additive Modeling	Real-world data sets served as the inspiration for the implementation	Although two buildings were used in the simulations, the number of data from building one is restricted
^9^	2019	Case-Based Reasoning	SCADA system allows flexibility for real-time access to data, as well as archiving	By increasing the agents' number, there is a possibility that its performance will suffer
^10^	2020	PSO + FOG computing	The PSO technique decreases task waiting time, latency, and network bandwidth	It is possible to experiment with different parameters, such as total electrical demand
^11^	2019	Graph theory + IoT	Less computational time due to fast organizing data as it can quickly locate the shortest path and the nodes' neighbors	Poor display of results
^12^	2020	Hadoop is used to connect to the cloud	Hadoop's distributed architecture for processing and storing data allows for the rapid processing of massive datasets	Kerberos authentication raises severe concerns about the absence of storage and network-level encryption
^13^	2020	Readings taken both indoors and outside are sent to the cloud for subsequent analysis and decision-making on energy efficiency	Generalized model may apply to any building in Europe	Security threat in the cloud
^14^	2019	Smart plug data is analyzed in the cloud	The cloud can track energy consumption in real-time through the smart plug	They did not specify the building type under study
^16^	2020	Fuzzy Fog Model	User data can be evaluated locally, ensuring the data's privacy	Data transmission bandwidth can be prohibitively expensive
^17^	2019	Game Theory Fog Model	The proposed framework for assessing decision-making in such circumstances as are encountered	Every participant is required to be aware of the other participants' cost functions
^18^	2019	Cloud Fog TCP/IP Model	The data can be processed at the data source that is geographically located closest to the user	Costs and efforts are incurred when registering a domain name
^19^	2019	Open System Interconnection–based smart meter design	The layer interfaces are consistent	The used communication technology supports one-way communication without acknowledgment
^20^	2020	Fuzzy logic for minimum pricing energy cost	Lower hardware requirements	The used hardware may suffer from measurement errors
^21^	2020	K-means clustering groups energy-using buildings	The k-means clustering algorithm is both quick and economical in terms of the amount of computing it requires	If the input data is of varying sizes, it nevertheless generates clusters of the same size

In this paper, an EMS for smart buildings is proposed. It depends on the coalition method for the optimal distribution of the surplus energy in the smart building on the deficient energy buildings. Shapley-based game theory is applied to obtain an optimal distribution of the surplus energy to the deficient energy buildings based on the required energy demand, achieving minimum energy waste and ensuring the generation-demand balance. The central management system is proposed for the one building based on cloud computing as it provides better scalability, centralized management, and easy-to-use and high performance. However, for the whole smart city, fog computing is applied, whereas it provides better scalability for handling large, distributed data sets. A communication channel-based Wi-Fi protocol transfers data and information between the smart buildings and the central EMS unit. A fog platform with storage capabilities is used for data handling and processing. A smart meter is applied to link the smart buildings with the central EMS unit. Two scenarios are implemented regarding the difference between the total energy surplus and deficiency. The first scenario is applying the coalition game theory for the optimal surplus energy allocation on the deficient energy buildings when the total energy surplus is lower than the amount of the total energy deficient. However, suppose the total energy surplus exceeds the required energy deficiency. In that case, the one-to-one relationship between the buildings with available energy surplus and others with energy-deficient is performed. The extra surplus energy is stored for further usage. The proposed method is verified using a hypothetical smart city including 10 smart buildings in MATLAB program. The results of each scenario are obtained, and a comparison between the result with/without applying the proposed method is introduced. Also, the performance of the fog platform is evaluated according to the run time, delay time, and memory size.

The rest of the work is organized as follows; section “Problem statement” presents the problem statement, and the full description and modeling for the proposed system are explained in section “Problem description and system modeling”. Game theory for collation strategy is explained in section “Game theory for collation strategy”. In section “Results and discussion”, the results of the simulation and the discussion are covered. The last part is the conclusion, which may be found in section “Conclusions and future scope”.

## Problem statement

One of the main goals of an EMS is to meet energy demand effectively and efficiently. It is necessary to regulate energy dispatch and systematically analyze power quality for a well-functioning energy system. The purpose of the EMS is to produce and distribute electrical energy in accordance with demand, preventing energy shortage/surplus during periods of less/large demanded power than generated power (off-peak/on-peak hours). Surplus energy is in some way stored using energy storage devices; nevertheless, power shortage is always a concern and cannot be stored. As a result, it is preferable to provide energy after predicting the available generation and demand^22^. Authors in^23^ suggested a load prediction system, and there is a disparity between expected and consumed energy. However, energy demand is always stochastic, the main cause of the imbalance between power supply and demand. As either a result, electricity is wasted and scarce. Work in^24^ suggested an SMGs framework to handle such a situation, where the EMS, after acquiring more energy from other sources, fills the demand for the deficit loads. Moreover, some buildings may purchase energy in bulk and cannot consume all energy within a time duration. The EMS can handle this issue by coordinating among the buildings where excess energy may be dispersed among power-deficient buildings through coordination. This is cost-effective for buildings with insufficient electricity and lessens the utility's burden.

Many more functionalities are included in the aforementioned EMS. Its installation individually becomes troublesome regarding flexibility, scalability, and cost. Furthermore, the products have tight guidelines for managing, controlling, and processing the number of linked devices. Moreover, even if consumers wish to access part of the features, they must pay for the entire package^25^.

Different energy management strategies are proposed based on centralized and distributed algorithms. Distributed algorithms are designed to be run on a network of interconnected devices rather than on a single, central computer. This makes them well-suited for studying energy management in smart buildings, which often involve a large number of devices and sensors distributed across a building or campus. Distributed algorithms and peer-to-peer (P2P) mechanisms in energy trading can provide several advantages, including decentralization of energy trading, improved security, increased transparency, reduced transaction costs, enhanced flexibility and resilience, and enabled the prosumers to produce electricity using renewable energy sources^26–28^. However, there are many challenges using distributed algorithms and peer-to-peer mechanisms, such as complexity in design and implementation, inadequate response, vulnerability to security threats, lack of control, and some nodes may have more resources or influence than others, leading to inequality and potential issues with fairness^26–30^. The fog server or a fog computing device is located at the network's edge, closer to the devices or sensors that generate data. A fog server is used to facilitate communication and coordination between different nodes in the network. The main advantages of fog computing include latency reduction, increased scalability, improved security, cost reduction, handling large amounts of data, improved the quality of service (QoS), and enabling IoT applications^31,32^. However, whether a fog server is suitable for this purpose would depend on the specific requirements and constraints of the peer-to-peer trading system. Factors such as the volume and complexity of the trading data, the need for real-time processing, and the security and privacy requirements of the system would all need to be considered when determining the most appropriate computing architecture for the system.

## Problem description and system modeling

**Figure 1:**
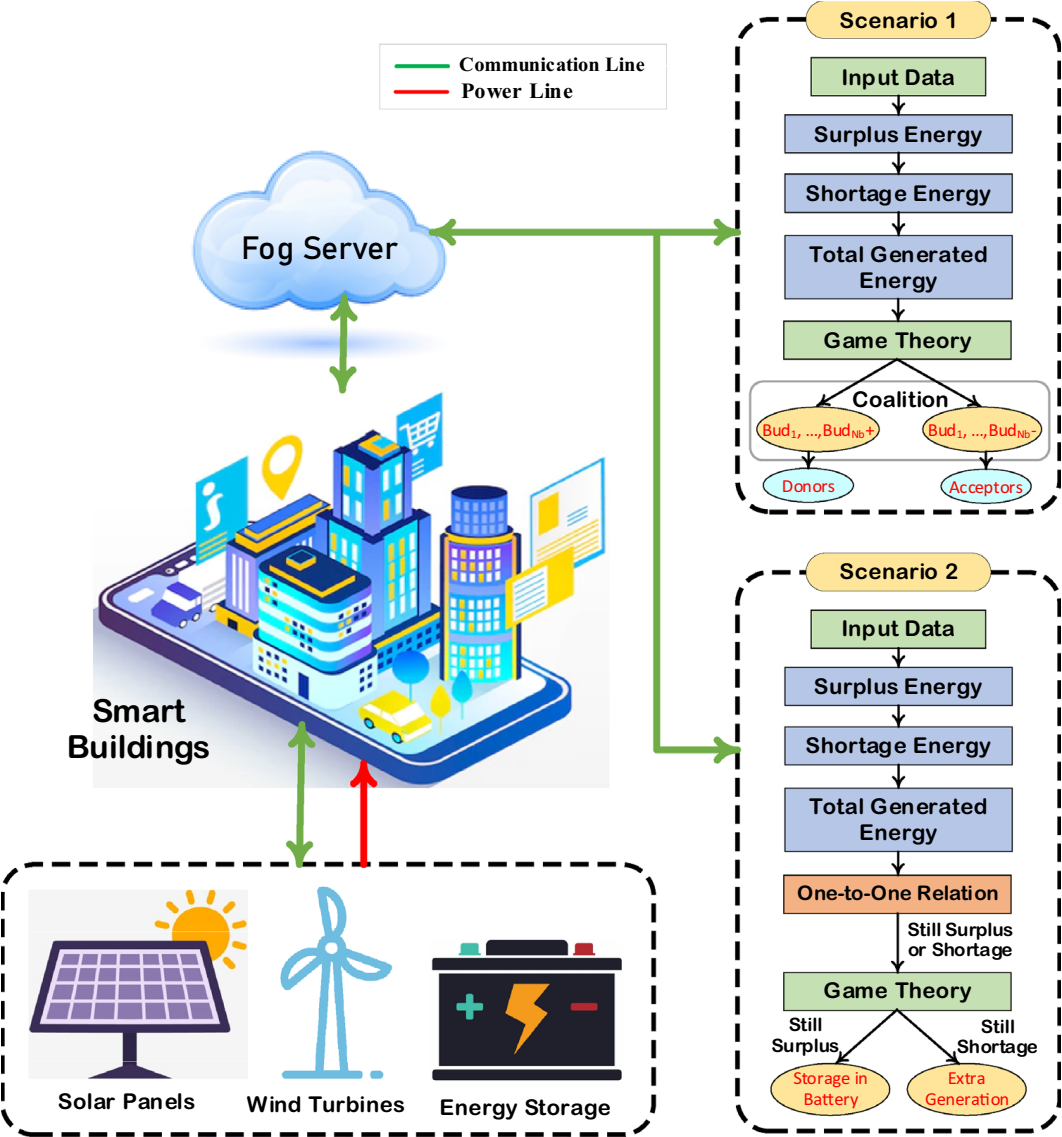
Model of SEM with fog computing and smart metering strategy.

This section addresses the model for sharing energy between customers using coordination for a surplus or power shortage of buildings. Figure 1 provides a structured description of the suggested system model, data exchange, and energy transfer. This model is made up of a smart building with number of buildings $({N}_{b})$, which is connected to local SMG (i.e., power suppliers) via fog. The fog is embedded with an energy control center used as a platform to handle energy management matters. The energy service provider collects data on the present load customers are utilizing and their demand for the upcoming term. In the same way, it shares basic data like the price of electricity and generated energy limits through the fog. Inside the building, the service provider guarantees that electricity from the SMG is delivered to the buildings. The communication medium among buildings and fog could be Bluetooth, Ethernet, Wireless, etc., based on the specifications of data transfer protocols. A Wi-Fi-embedded smart meter acts as a communication channel between buildings and proposed game theory-based EMS placed in fog.

SMGs gather energy consumption data and plan for the day's power generation. Additionally, it maintains a balance between power demand and generation by directly demand side management (DSM) or extra generation. The EMS will gather data from users who need extra energy and those who have surplus energy and make decisions based on that knowledge. Coordination is enabled between the ${N}_{b}^{-}$ buildings with a shortage of energy and ${N}_{b}^{+}$ energy surplus buildings. Moreover, a coalition of ${N}_{b}^{-}$ shortage energy buildings will be formed to distribute the shortage power unevenly in accordance with demand, enabling service providers to balance power generation and demand and prevent energy waste.

The controller will collect data from customers who need more demand than his generated power from his renewable resources by taking energy from other customers with extra energy. So, customers should coordinate with each other to make the balance between the ${N}_{b}^{+}$ customers who have extra generated energy and ${N}_{b}^{-}$ customers who have extra load demand and minus energy.

Based on the game theory and to enhance EMS by minimizing the power wasted and rescuing electricity cost, the coordination problem is modeled as a social network issue. A three-tuple is used in a game of coalition creation $\left(\aleph , \vartheta ,\xi \right)$, where $\aleph =\left\{1, 2,\dots , {N}_{b}^{-}\right\}$ is a group of ${N}_{b}^{-}$ players that attempt to cooperate to strengthen their positions in the games (i.e., $\aleph =$ ${BE}_{{N}_{b}^{-}}^{-}$, where ${BE}^{-}$ is the building with a shortage of energy), $\vartheta = 2^{{N_{b}^{ - } }} \to {\Re }$ is a characteristic function that specifies subsets of players and is depicted as a real number to each coalition $S$, where $S\subseteq \aleph$ is the overall payoff (surplus energy) that each coalition has achieved. $\xi$ is a vector that depicts the payoffs received by players from the value $\vartheta$.

In this work, each energy shortage building ${BE}_{j}^{-}$ is treated as a player, and it searches for another energy deficient ${BE}_{k}^{-}$ (i.e., $j,k\in \aleph$ and $j\ne k$) to form a coalition to maximize the payoff in terms of extra power for itself and other players. Two groups make up this coalition, the donors (contributors ${BE}_{{N}_{b}^{+}}$) and the acceptors (${BE}_{{N}_{b}^{-}}^{-}$). The total surplus electricity (${BE}_{Total}^{+}$) is the total available payoff by ${N}_{b}^{+}$ buildings that have extra generated power $({BE}_{i}^{+})$ which is given as:

1 $${BE}_{Total}^{+}={\sum }_{i=1}^{{N}_{b}^{+}}{BE}_{i}^{+}$$

By distributing ${BE}_{Total}^{+}$ among the buildings have an energy shortage ${BE}_{{N}_{b}^{-}}^{-}$ according to the nature of loads and price of energy, which leads to reducing the wasted electricity $(WE)$. As a result, the difference between total distributed energy (${DisE}_{Total}$) for ${N}_{b}^{-}$ customers and ${BE}_{Total}^{+}$ can be minimum. The objectives under discussion are expressed mathematically as follows:

$$Minimize (WE)$$

Subject to

2 $$min(\Delta {BE}_{Total}^{+}, {\Delta DisE}_{Total})$$

where

3 $${DisE}_{Total}=\sum_{i=1}^{{N}_{SE}^{-}}{DisE}_{i}$$

where $\Delta {BE}_{Total}^{+}$ is the total change in surplus electricity and ${\Delta DisE}_{Total}$ is the total change in distributed energy. ${BE}_{Total}^{+}$ is represented as $WE$. As previously stated, the ${BE}_{Total}^{+}$ is distributed among ${BE}_{{N}_{b}^{-}}^{-}$ based on their energy demand $\left({LD}_{{N}_{b}^{-}}\right)$. The total power shortage for ${N}_{b}^{-}$ customers is expressed as:

4 $${LD}_{Total}={\sum }_{i=1}^{{N}_{b}^{-}}{LD}_{i}$$

where ${\mathrm{LD}}_{\mathrm{Total}}$ is the total shortage power of all loads which maybe more than, less than, or equal to ${\mathrm{BE}}_{\mathrm{Total}}^{+}$.The difference $\left({D}_{T}\right)$ between total surplus power $\left({BE}_{Total}^{+}\right)$ and total shortage power $\left({\mathrm{LD}}_{\mathrm{Total}}\right)$ can be expressed as:

5 $${D}_{T}={BE}_{Total}^{+}- {LD}_{Total}$$

where

$${D}_{T}=\left\{ \begin{array}{cc}<0, & if { BE}_{Total}^{+}< {LD}_{Total} \\ =0, & if {BE}_{Total}^{+}< {LD}_{Total} \\ >0, & if {BE}_{Total}^{+}< {LD}_{Total} \end{array}\right.$$

It is crucial to note that the suggested approach is cost-effective for both SMGs and consumers. When a sudden load demand increases, service providers must supply extra energy, and customers must pay an additional bill. On the other side, if some buildings reduce their run-time demands without being aware of the extra power generated, there is a chance that they would waste electricity. Several scenarios may be implemented to share the excess energy among the units with insufficient energy.

In this study, two scenarios are taken into account. In scenario 1, collaboration among buildings with excess power and those with insufficient power is achieved through coalitions. Two groups are established, one including the energy-surplus buildings and the other containing the energy-deficient units. The entire extra energy is distributed among the buildings with a power shortage after being coordinated. The remaining extra energy is neither produced nor stored in this scenario. In contrast, no coalition is needed to create coordination between a power surplus and a deficient power building in scenario 2. Additionally, extra energy is stored and will be produced if necessary. The following is a complete overview of these two scenarios:

### Scenario 1

It formed a coalition with coordination between customers who need extra demand and other customers who have extra generation. The surplus energy is distributed to the power-shortage buildings after coordination. Extra energy is not generated or saved in this scenario. ${BE}_{Total}^{+}$ is distributed through all of the ${BE}^{-}$ by making a coalition in the case if ${LD}_{Total}>{BE}_{Total}^{+}$. Here each ${BE}^{-}$ receives a payoff based on the load demand of consumers, i.e., if ${BE}_{a}^{-}$ demand is greater than ${BE}_{b}^{-}$ then ${BE}_{a}^{-}$ give extra power in this scenario (i.e., ${BE}_{i}^{+}={LD}_{j}$). This is represented mathematically as:

6 $$\left\{\begin{array}{c}{DisE}_{a}< {DisE}_{b} , if {BE}_{a}^{-}< {BE}_{b}^{-}\\ {DisE}_{a}\ge {DisE}_{b} , if {BE}_{a}^{-} \ge {BE}_{b}^{-} \end{array}\right.$$

where $a,b\in {N}_{b}^{-}$ and $a\ne b$. In this case, the EMS permits the coalition of energy-shortage buildings daily.

### Scenario 2

When ${LD}_{Total}\le {BE}_{Total}^{+}$, the EMS checks the condition; if a building has an extra energy ${BE}_{i}^{+}$ equals any ${LD}_{j}$, regardless of the needs of other buildings, the EMS establishes the coordination between these two buildings. Additionally, extra energy is stored and will be used if necessary. However, if necessary, it will permit the coalition among the ${BE}_{l}^{-}$ (i.e., $l \ne j$) as stated in (6). This is represented mathematically as:

7 $$\left\{\begin{array}{c}{DisE}_{j}={BE}_{i}^{+}, if{ BE}_{j}^{-}={BE}_{i}^{+} \\ {DisE}_{a}<{DisE}_{b} , if{ BE}_{a}^{-}<{BE}_{b}^{-} \\ {DisE}_{a}\ge {DisE}_{b} , if{ BE}_{a}^{-}\ge {BE}_{b}^{-} \end{array}\right.$$

where $i\in {N}_{b}^{+}$, $j\in {N}_{b}^{-}$, $a, b\in {N}_{SE}^{-}$ and $a\ne b$. Additionally, in this case, the EMS will allow building coordination before the start of the day.

## Game theory for collation strategy

Fog computing and game theory are implemented together in the proposed energy management system as they have several advantages^33,34^. Fog computing enables real-time data processing at the network's edge, which can be used to make decisions about energy management in actual time. An equilibrium that maximizes the overall efficiency of the energy system can be found by using game theory to model the interactions between the various players in the energy system. Also, by simulating the interactions between various energy sources and ensuring a steady supply of energy, fog computing and game theory can be used to increase the energy system's resilience. Finally, combining game theory and fog computing can lower the costs related to energy management. The game theory minimizes wasted energy ($WE$) while preserving a balance between supply and demand. For collation game $\left(\aleph , \vartheta , \xi \right)$, the payoff of each building (player)$j$ will be estimated based on the Shapley value rule^35^. This for each ${BE}_{j}^{-}$ identifies as (${\xi \left(f\right)}_{j}$) and is determined as:

8 $${\xi \left(\vartheta \right)}_{j}=\sum_{S\subseteq \aleph \backslash \{j\}}\frac{\left|S\right|!(\aleph -\left|S\right|-1)!}{\aleph !}(\vartheta (S\cup \{j\}-\vartheta (S)).$$

Each building $j$ participating in the game is treated as a player for the sake of energy management, and the overall profit is used as the ${BE}_{Total}^{+}$. Formally, the distribution of ${BE}_{Total}^{+}$ according to (5) is expressed as follows:

9 $${DisE}_{j}=\left\{\begin{array}{c}{LD}_{i}, if{LD}_{Total}\le {BE}_{Total}^{+}\\ {\xi \left(\vartheta \right)}_{j}, if{LD}_{Total}>{BE}_{Total}^{+}\end{array}\right.$$

where at the end of the case ${LD}_{Total}> {BE}_{Total}^{+}$, the ${EDis}_{Total}$ will be less than ${LD}_{Total}$ and ${EDis}_{Total}=\sum_{j=1}^{{N}_{b}^{-}}{\xi \left(\vartheta \right)}_{j}$

### Algorithm for Scenario 1

The power is delivered according to demand for the condition ${LD}_{Total}\le {BE}_{Total}^{+}$. However, in the case of ${LD}_{Total}>{BE}_{Total}^{+}$, an effective and immediate implementation mechanism is required to aid in the equitable distribution of ${BE}_{Total}^{+}$ among ${BE}_{{N}_{b}^{-}}^{-}$. Fair in the sense that more energy will be delivered to a building if it needs more than the average amount.
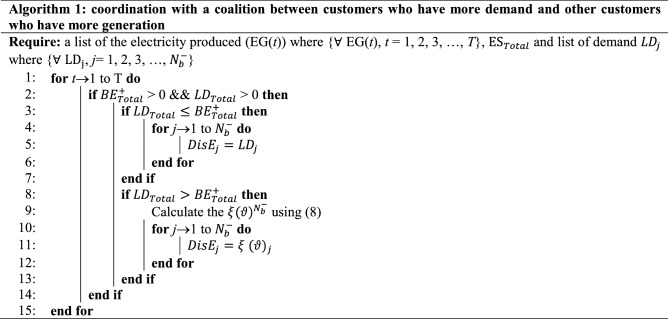


### Algorithm for Scenario 2

**Figure 2:**
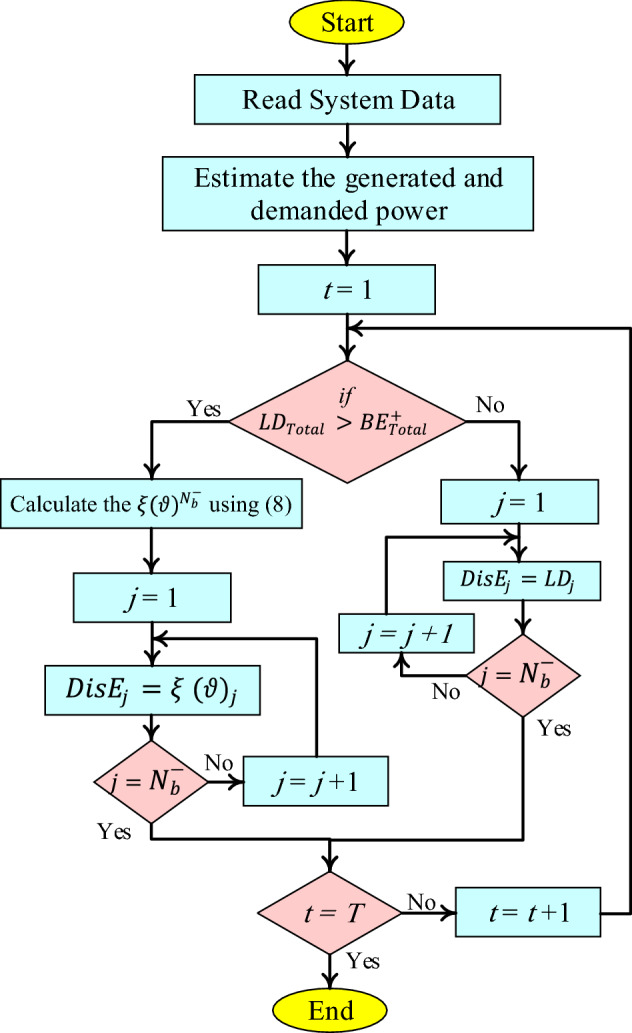
The flowchart for the proposed EMS algorithm in SMGs.


For the requirement ${LD}_{Total}\le {BE}_{Total}^{+}$, the EMS achieves coordination for the buildings by establishing one-to-one relations between ${BE}_{i}^{+}$ and ${BE}_{j}^{-}$, in this case ${BE}_{i}^{+}$ equals ${LD}_{j}$. This connection is formed by adjusting basic social network behavior, in which a person coordinates with others based on their needs. Otherwise, if ${BE}_{l}^{-}>0$, a coalition of power shortage among buildings is formed, and surplus energy is distributed based on (8). The proposed EMS algorithm flowchart for smart buildings is shown in Fig. 2.

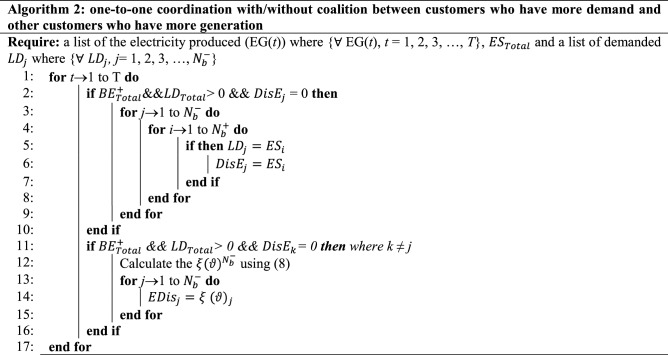


Coalition game theory has some advantages^36–38^, such as enabling the simulation of complex interactions between groups of players, which is helpful when there are disparate motivations or goals among the players. Coalition game theory can shed light on how decisions are made in groups and how these decisions affect the overall result of a game by observing the behavior of player groups. It can be used to find stable solutions, such as the bargaining set or the core, that can be used to forecast how players will act in groups. Coalition game theory can be used to examine the power relationships between various groups of players and comprehend how these relationships affect how a game turns out. Finally, it can be used to model, examine, and design the circumstances in which cooperation is possible.

## Results and discussion

**Figure 3:**
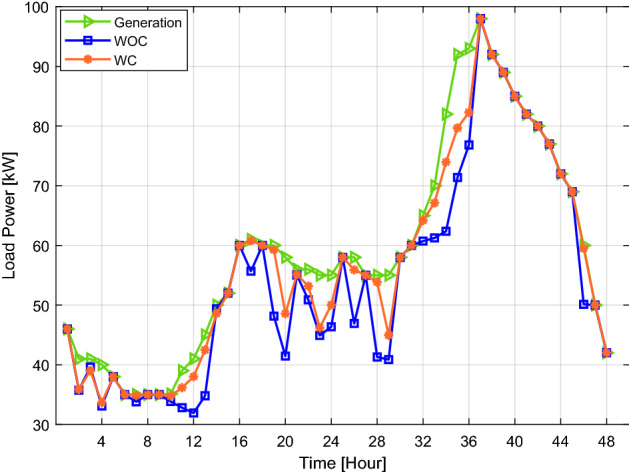
The generated power and energy consumption for coordination and without coordination.

The simulation is done for the above scenarios; the results are discussed in this section. For the sake of the simulation, a smart city of ten buildings is under consideration. Furthermore, we do all management calculations on the fog, allowing us to coordinate between extra and shortage energy buildings. As well as make a coalition for buildings that have a shortage of energy. The system's real-time data has been taken from^39^, which provides the pattern of load demand in a day during the different seasons of the year. This facilitates data collection on the average electrical load profile consumed by users. This data is obtained and updated every minute; 48 h time slots are seen in Fig. 3. The proposed EMS aims to reduce wasted energy by establishing coordination among customers with surplus energy and a shortage in energy. A smart meter will link all of the participant's devices to the EMS located on fog at 11:55 p.m., gathering data at the start of the day.

### Results analysis for Scenario 1

**Figure 4:**
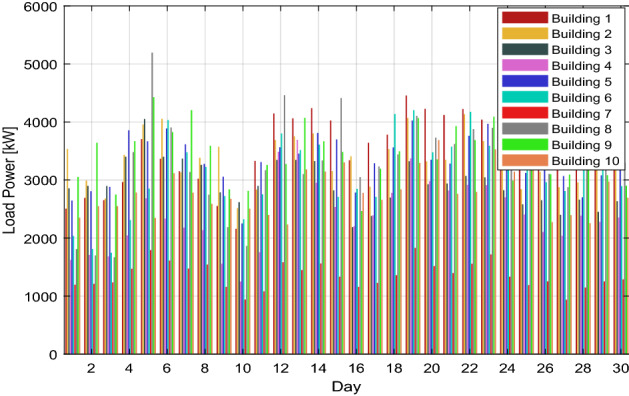
The electricity load profile of 10 buildings over the month.

In this scenario, consider the consumed energy as the produced energy due to the service provider forecasting the load demand energy. However, as illustrated in Fig. 4, the customers' energy demands are stochastic and subject to change at any moment of the day. Some users could need more energy, while others would desire a lower load profile than they had the day before.

**Figure 5:**
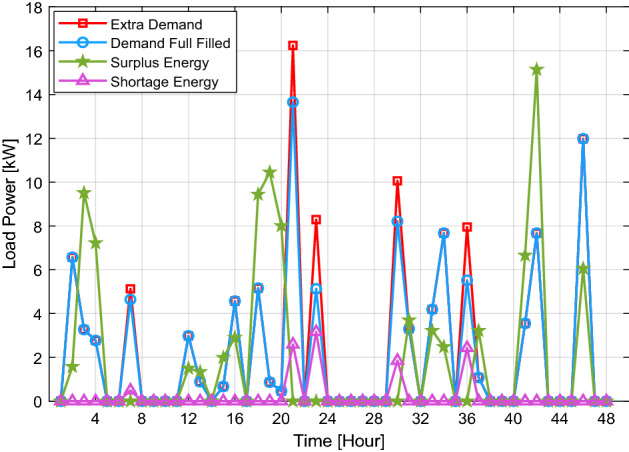
The building's extra energy requirement, as well as the excess energy with and without coordination.

Figure 3 depicts the generated and utilized electrical power for 10 buildings with coordination (WC) and without coordination (WOC). As shown in Fig. 3, if the buildings do not coordinate with each other, the energy generated may be wasted even if just one building lowers its power usage. At the same time, some buildings can suffer when they need additional energy. The difference between the energy produced and utilized at 10:00 p.m. is 10 kW for WC and 17 kW for WOC. Additionally, Fig. 5 displays the excess energy used by the buildings (deficit energy WOC), surplus energy WOC, satisfied demand (shortage energy WC), and surplus energy WC through various periods. The hourly details of these energies are presented in Table 2. The second column represents the amount of extra energy in some buildings, and the third column shows the amount of covered energy from this extra energy distributed to buildings that need energy. The fourth and fifth columns show the amount of energy deficit or surplus after EMS.

**Table 2:** Hourly extra, demanded, surplus, and shortage energy.

Hour	Extra demand	Demand full filled	Surplus energy	Shortage energy
1	0.0	0.0	0.0	0.0
2	6.6	6.6	1.6	0.0
3	3.3	3.3	9.5	0.0
4	2.8	2.8	7.2	0.0
5	0.0	0.0	0.0	0.0
6	0.0	0.0	0.0	0.0
7	5.1	4.6	0.0	0.5
8	0.0	0.0	0.0	0.0
9	0.0	0.0	0.0	0.0
10	0.0	0.0	0.0	0.0
11	0.0	0.0	0.0	0.0
12	3.0	3.0	1.5	0.0
13	0.9	0.9	1.3	0.0
14	0.0	0.0	0.0	0.0
15	0.7	0.7	2.0	0.0
16	4.6	4.6	2.9	0.0
17	0.0	0.0	0.0	0.0
18	5.2	5.2	9.4	0.0
19	0.9	0.9	10.5	0.0
20	0.5	0.5	8.0	0.0
21	16.2	13.7	0.0	2.6
22	0.0	0.0	0.0	0.0
23	8.3	5.1	0.0	3.2
24	0.0	0.0	0.0	0.0
25	0.0	0.0	0.0	0.0
26	0.0	0.0	0.0	0.0
27	0.0	0.0	0.0	0.0
28	0.0	0.0	0.0	0.0
29	0.0	0.0	0.0	0.0
30	10.1	8.2	0.0	1.9
31	3.3	3.3	3.7	0.0
32	0.0	0.0	0.0	0.0
33	4.2	4.2	3.2	0.0
34	7.7	7.7	2.5	0.0
35	0.0	0.0	0.0	0.0
36	8.0	5.5	0.0	2.4
37	1.1	1.1	3.2	0.0
38	0.0	0.0	0.0	0.0
39	0.0	0.0	0.0	0.0
40	0.0	0.0	0.0	0.0
41	3.6	3.6	6.7	0.0
42	7.7	7.7	15.1	0.0
43	0.0	0.0	0.0	0.0
44	0.0	0.0	0.0	0.0
45	0.0	0.0	0.0	0.0
46	12.0	12.0	6.1	0.0
47	0.0	0.0	0.0	0.0
48	0.0	0.0	0.0	0.0

Figure 5 shows the building's extra energy requirement and excess energy. However, the difference in the case of WOC is about 12 kW. In addition, over various periods, excess energy is needed for the buildings with less energy. Figure 5 shows the building's extra energy requirement, and the excess energy (with and without coordination), extra energy without coordination, fulfilled (demand minus energy with coordination), and extra energy with coordination are depicted in Fig. 5.

**Figure 6:**
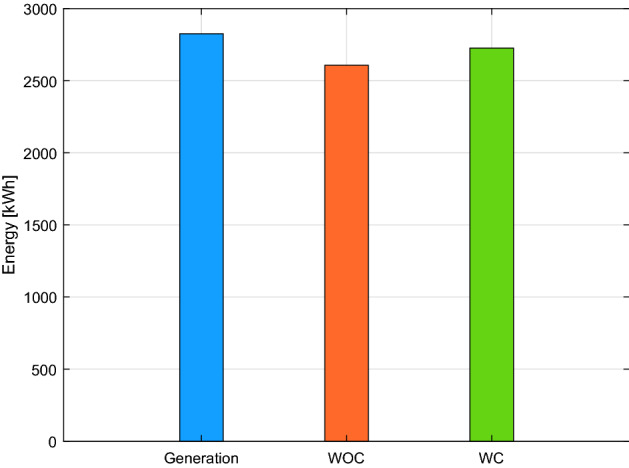
Generation, energy consumption with and without coordination throughout the day.

**Figure 7:**
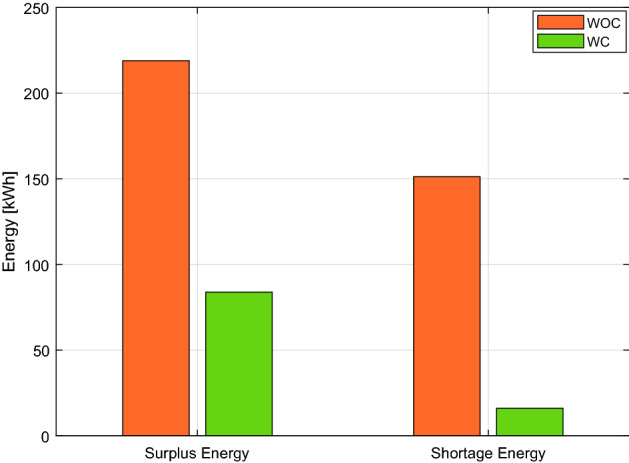
Minus and extra energy throughout the day.

Figure 6 depicts the total effect on power consumption with and without the coalition. A total of 2865.7 kW of energy is generated; however, if a coalition is not formed among the buildings, 227.45 kW of electricity is wasted. As demonstrated in Fig. 3, 86.43 kW of power is squandered when a coalition is formed. Furthermore, Fig. 7 shows that buildings are affected by WOC because 141 kW more electricity is required than usual at various intervals. However, the 18.85 kW slack in WC buildings is insignificant. In the scenario where generation is more than demand, as shown in Fig. 7, energy might be wasted. Consumers of electricity may also suffer if the utility forbids them to use the energy beyond a set threshold and the consumer needs more power. Our suggested plan successfully uses a coalition to balance generation and demand while combining the additional demand in emergencies.

### Results analysis for Scenario 2

**Figure 8:**
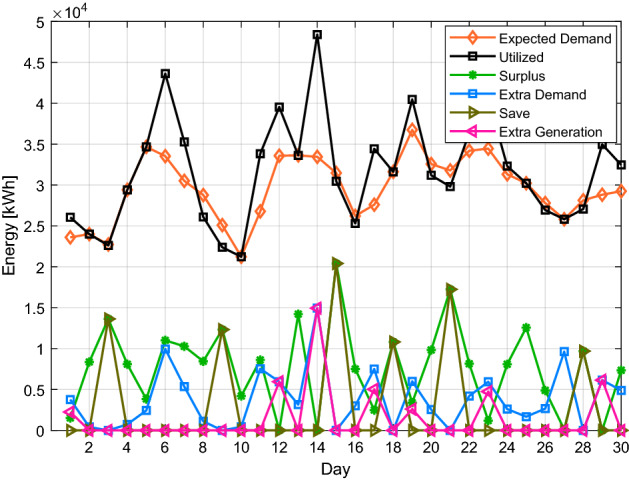
Demand and surplus generated power for a month.

**Figure 9:**
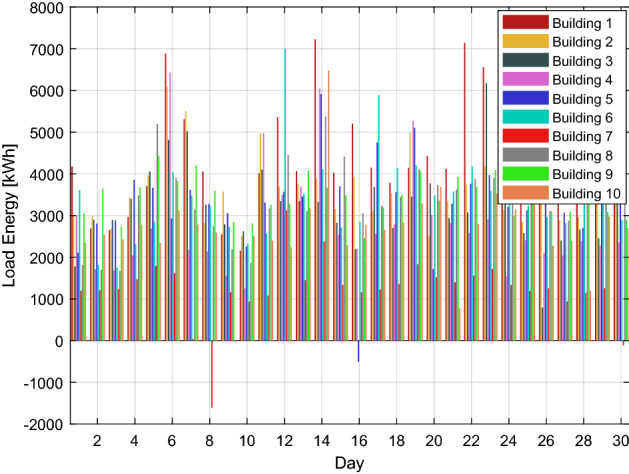
Load demand pattern after make coordination between buildings.

**Figure 10:**
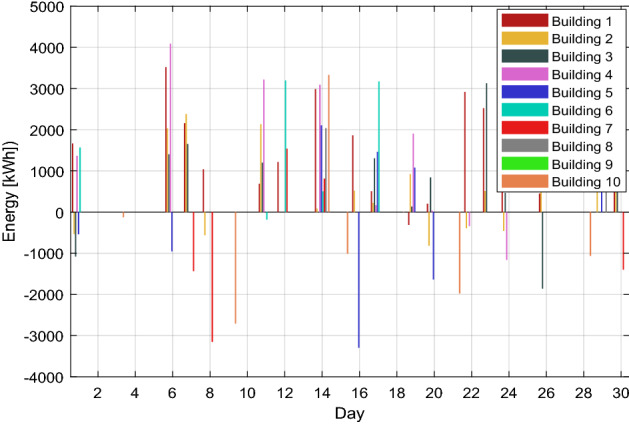
Extra and shortage of energy throughout the month for each building.

**Figure 11:**
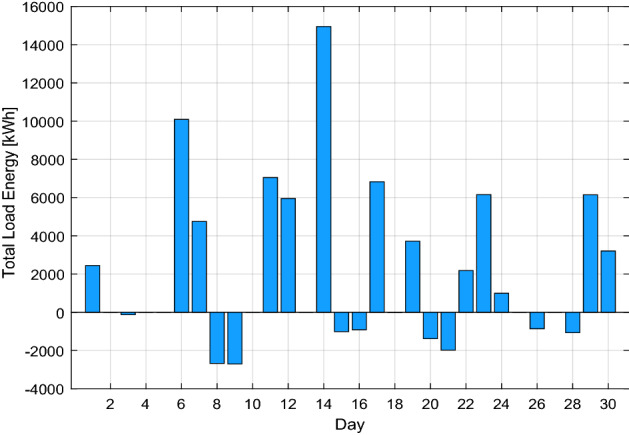
Total energy requirement for all buildings during the month.

Algorithm 2 will make decisions based on demand and excess energy. Surplus energy may be produced from the local MG if necessary; moreover, surplus energy is stored, as seen in Fig. 8. This excess energy stored can be used later. While in scenario 1, even after coalition energy is spent, surplus energy is not produced nor stored as a result. Figure 9 shows a noticeable change in the energy profile after a coalition of surplus energy buildings and their cooperation with a group of energy shortage units. Figure 10 shows a detailed representation depiction of power shortage and surplus buildings. As shown in Table 3, the negative numbers in this graph reflect an energy deficit, while the positive ones represent an energy surplus. The deficient energy in this scenario is the additional demanded energy that decreased to a minimum value when coordinating with the coalition is established. As demonstrated in Fig. 8, the likelihood of wasted energy increases if coordination is not created. Also, the probability of energy waste will increase if coordination is not created. Figure 11 shows the difference between the fed daily load and the unfed demand. The positive direction represents the energy consumed by the 10 buildings, while the negative direction in this figure reflects an energy shortage.

**Table 3:** The surplus and shortage of energy of each building throughout the month.

Day/building	1	2	3	4	5	6	7	8	9	10
1	1665.1	− 535.6	− 1079.6	1365.5	− 537.9	1569.1	0.0	0.0	0.0	0.0
2	0.0	0.0	0.0	0.0	0.0	0.0	0.0	0.0	0.0	0.0
3	0.0	0.0	0.0	0.0	0.0	0.0	0.0	0.0	0.0	− 124.1
4	0.0	0.0	0.0	0.0	0.0	0.0	0.0	0.0	0.0	0.0
5	0.0	0.0	0.0	0.0	0.0	0.0	0.0	0.0	0.0	0.0
6	3519.0	2032.3	1402.9	4090.7	− 955.8	0.0	0.0	0.0	0.0	0.0
7	2158.2	2380.2	1652.3	0.0	0.0	0.0	− 1436.4	0.0	0.0	0.0
8	1036.4	− 563.0	0.0	0.0	0.0	0.0	− 3151.4	0.0	0.0	0.0
9	0.0	0.0	0.0	0.0	0.0	0.0	0.0	0.0	0.0	-2705.9
10	0.0	0.0	0.0	0.0	0.0	0.0	0.0	0.0	0.0	0.0
11	687.4	2132.0	1198.3	3215.6	0.0	-183.6	0.0	0.0	0.0	0.0
12	1216.6	0.0	0.0	0.0	0.0	3197.1	1539.9	0.0	0.0	0.0
13	0.0	0.0	0.0	0.0	0.0	0.0	0.0	0.0	0.0	0.0
14	2987.7	83.0	0.0	3091.3	2105.5	502.1	811.7	2035.6	0.0	3331.0
15	0.0	0.0	0.0	0.0	0.0	0.0	0.0	0.0	0.0	− 1010.8
16	1860.6	517.9	0.0	0.0	− 3292.5	0.0	0.0	0.0	0.0	0.0
17	506.0	220.4	1305.9	164.9	1461.0	3172.1	0.0	0.0	0.0	0.0
18	0.0	0.0	0.0	0.0	0.0	0.0	0.0	0.0	0.0	-6.1
19	− 311.5	919.0	131.8	1901.8	1077.7	0.0	0.0	0.0	0.0	0.0
20	201.1	− 818.4	841.4	33.1	− 1636.0	0.0	0.0	0.0	0.0	0.0
21	0.0	0.0	0.0	0.0	0.0	0.0	0.0	0.0	0.0	− 1977.9
22	2915.3	− 391.8	0.0	− 338.9	0.0	0.0	0.0	0.0	0.0	0.0
23	2519.9	509.0	3126.9	0.0	0.0	0.0	0.0	0.0	0.0	0.0
24	2146.0	− 457.7	466.0	− 1159.6	0.0	0.0	0.0	0.0	0.0	0.0
25	0.0	0.0	0.0	0.0	0.0	0.0	0.0	0.0	0.0	0.0
26	447.6	551.5	− 1860.8	0.0	0.0	0.0	0.0	0.0	0.0	0.0
27	0.0	0.0	0.0	0.0	0.0	0.0	0.0	0.0	0.0	0.0
28	0.0	0.0	0.0	0.0	0.0	0.0	0.0	0.0	0.0	− 1064.4
29	0.0	2713.9	0.0	0.0	1633.5	0.0	0.0	1800.8	0.0	0.0
30	1592.6	1519.3	1496.2	0.0	0.0	0.0	− 1398.8	0.0	0.0	0.0

According to real-time data shown in Figs. 3 and 4, it is easy to imagine that the load profile of each customer has a unique energy requirement. It is conceivable that the load profile of the customers varies daily. Additionally, it is determined from the outcomes of scenarios 1 and 2 that a maximum amount of energy may be lost if energy usage is not adequately monitored. A suitable medium is needed to monitor and make choices quickly and with the least delay.

The literature review reveals that the game theory technique is one of the most well-known coordinating methods. However, as the number of players grows, so does its complexity concerning time and space. Several occurrences may cause a bottleneck in the network. An EMS is utilized over fog computing to prevent such a scenario and maintain the system functioning efficiently.

### Fog computing performance evaluation

**Figure 12:**
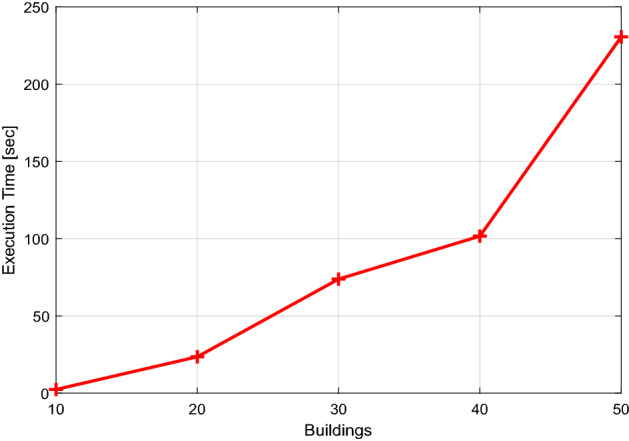
The run-time on the fog server.

**Figure 13:**
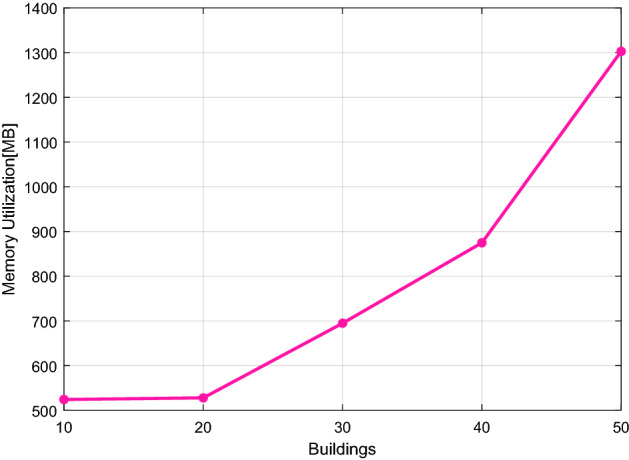
Memory space utilization on the fog server.

**Figure 14:**
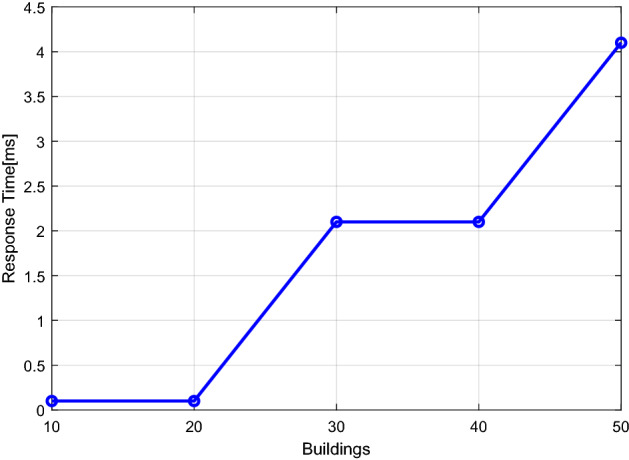
The data's estimation of the network response delay time.

For scenario 2, a program's execution time, memory needs, and network response time are determined to validate and investigate the complexity of the application. Figures 12 and 13 provide a graphical depiction of the execution time and memory utilization. These numbers show that program execution time and memory use increase as the number of buildings participating in the coalition rises. The minimum execution time for ten buildings is 2.40 s, and for 50 buildings, it is 230.60 s. Additionally, extra memory is needed to carry out the computation; as seen in Fig. 12, ten units require 524.24 MB of memory, while 50 buildings demand up to 1303 MB. This conversation demonstrates the urgent need for a quick computational medium close to the network to prevent energy supply delays. Figure 14 studies the network response time; it is obvious from this figure that the response time is lengthy relative to the data.

The results findings in Figs. 12 and 13 and 14 show how an application's execution time and memory needs grow as the number of buildings rises. As a result, a platform is needed to eliminate service delays and have a fast reaction time, which may be achieved by putting the consumer application at the edge of the fog server. This server can decrease the delay of services and responds quickly.

## Conclusions and future scope

This paper proposed an EMS for a smart building based on coalition game theory for optimal allocation of the energy surplus on the energy-deficient buildings. Central EMS with a fog platform is designed for data receiving, processing, and decision-making. Communication channel-based Wi-Fi protocol transfers data, information, and control signals between the smart buildings and the EMS central unit. A smart meter interlinks between the smart buildings and the central fog-based EMS unit. Two scenarios are implemented based on the difference between the total energy surplus and deficient using MATLAB program for a smart city including 10 smart buildings. The results show the proposed EMS's ability to prevent up to 141 kW of electricity from being wasted daily. The future scope is applying the proposed method with considering the energy hub and the presence of load, generation, and communication uncertainties. Besides applying a control technique for packet loss reduction and enhancing the system security against any cyber-attacks.

## Data Availability

The datasets used and/or analyzed during the current study are available from the corresponding author on reasonable request.
